# Activation of Toll-like receptor 5 in microglia modulates their function and triggers neuronal injury

**DOI:** 10.1186/s40478-020-01031-3

**Published:** 2020-09-10

**Authors:** Masataka Ifuku, Lukas Hinkelmann, Leonard D. Kuhrt, Ibrahim E. Efe, Victor Kumbol, Alice Buonfiglioli, Christina Krüger, Philipp Jordan, Marcus Fulde, Mami Noda, Helmut Kettenmann, Seija Lehnardt

**Affiliations:** 1 Cellular Neuroscience, Max-Delbrück-Center for Molecular Medicine in the Helmholtz Association, Berlin, Germany; 2 Institute of Cell Biology and Neurobiology, Charité – Universitätsmedizin Berlin, corporate member of Freie Universität Berlin, Humboldt-Universität zu Berlin, and Berlin Institute of Health, Berlin, Germany; 3 Charité – Universitätsmedizin Berlin, corporate member of Freie Universität Berlin, Humboldt-Universität zu Berlin, and Berlin Institute of Health, Berlin, Germany; 4 Institute of Microbiology and Epizootics, Centre for Infection Medicine, Freie Universität Berlin, Berlin, Germany; 5 Laboratory of Pathophysiology, Graduate School of Pharmaceutical Sciences, Kyushu University, Fukuoka, Japan; 6 Department of Neurology, Charité – Universitätsmedizin Berlin, corporate member of Freie Universität Berlin, Humboldt-Universität zu Berlin, and Berlin Institute of Health, Berlin, Germany; 7 Present Address: Pharmaceutical Research Division, Takeda Pharmaceutical Company Limited, Fujisawa, Japan

**Keywords:** Toll-like receptor 5, Microglia, PI3K/Akt/mTORC1 signaling, Cytokines, Phagocytosis, Chemotaxis, Neuronal apoptosis

## Abstract

Microglia are the primary immune-competent cells of the central nervous system (CNS) and sense both pathogen- and host-derived factors through several receptor systems including the Toll-like receptor (TLR) family. Although TLR5 has previously been implicated in different CNS disorders including neurodegenerative diseases, its mode of action in the brain remained largely unexplored. We sought to determine the expression and functional consequences of TLR5 activation in the CNS. Quantitative real-time PCR and immunocytochemical analysis revealed that microglia is the major CNS cell type that constitutively expresses TLR5. Using *Tlr5*^−*/*−^ mice and inhibitory TLR5 antibody we found that activation of TLR5 in microglial cells by its agonist flagellin, a principal protein component of bacterial flagella, triggers their release of distinct inflammatory molecules, regulates chemotaxis, and increases their phagocytic activity. Furthermore, while TLR5 activation does not affect tumor growth in an ex vivo GL261 glioma mouse model, it triggers microglial accumulation and neuronal apoptosis in the cerebral cortex in vivo. TLR5-mediated microglial function involves the PI3K/Akt/mammalian target of rapamycin complex 1 (mTORC1) pathway, as specific inhibitors of this signaling pathway abolish microglial activation. Taken together, our findings establish TLR5 as a modulator of microglial function and indicate its contribution to inflammatory and injurious processes in the CNS.

## Background

Toll-like receptors (TLRs) are pattern recognition receptors that are activated by both pathogen- and host-derived, potentially damage-associated, molecular patterns [61]. Engagement of these membrane receptors in the brain can result in various outcomes, such as chronic inflammation, neuronal injury, either in a cell-autonomous or inflammation-associated fashion, or in regenerative processes [36, 40, 57]. Among the 13 members of the TLR family in mouse and human characterized to date, TLR5 is the only protein-binding TLR that is conserved in vertebrates from fish to mammals, and specifically recognizes flagellin, a fibrillar protein derived from bacteria [22, 31, 59]. Examples of flagellated bacteria are *Escherichia coli* and *Salmonella* Typhi, which both can enter the central nervous system (CNS), thereby causing meningitis and/or brain abscesses [1, 9, 19, 34]. Furthermore, recent studies identified TLR5 as being involved in different CNS disorders lacking a primarily pathogen-associated cause, including Alzheimer’s disease (AD), neuropathic pain, and cerebral ischemia [7, 24, 33, 64]. In peripheral immune cells such as dendritic cells and monocytes, and epithelial cells, flagellin-induced activation of TLR5 triggers PI3K signaling resulting in NF-κB activation, thereby modulating the innate immune response [13, 15, 47, 48, 56]. However, the TLR5-induced signaling cascade in the CNS is unresolved.

Microglia are the brain’s primary sensor for pathologic events and express all TLRs identified so far, including TLR5 [52]. Activation of distinct TLRs can affect diverse microglial functions including migration (via TLR7, [29]) and cytokine release (via TLR2, TLR4, TLR7, [5, 38, 40]). In the present study, we sought to systematically analyze the expression and function of TLR5 in the CNS. In particular, we focused on the molecular mechanisms and signaling pathway promoting microglial chemotaxis, phagocytosis, cytokine production, and interaction with glioma cells as a consequence of TLR5 activation in these cells. Furthermore, we analyzed whether microglial TLR5 activation may lead to neuronal injury.

## Methods

### Reagents

Purified recombinant flagellin from *Salmonella* Typhimurium (FLA-ST Ultrapure) and loxoribine were purchased from InvivoGen (San Diego, CA, USA). Lipopolysaccharide (LPS) was purchased from Enzo Life Sciences (Lörrach, Germany). LY294002 was obtained from Cell Signaling Technology (Danvers, MA, USA), while wortmannin and rapamycin were purchased from Sigma-Aldrich (St. Louis, MO, USA). Akt inhibitor IV was obtained from Calbiochem (San Diego, CA, USA). LY294002, Wortmannin, and rapamycin were solved in dimethyl sulfoxide (DMSO; Sigma-Aldrich, St. Louis, MO, USA). In all experiments using the inhibitors, DMSO-containing DMEM medium complete (see below; DMSO dilution at 1:1000 vol/vol) served as negative control. Anti-mTLR5 neutralizing IgG antibody was obtained from InvivoGen.

### Mice and cell lines

C57BL/6 (wild-type, WT) mice were obtained from the Charité animal facility, Berlin, Germany, or purchased from Charles River Laboratory (Wilmington, MA, USA). *Tlr5*^−*/*−^ (B6.129S1-Tlr5^*tm1Flv*^/J (Tlr5, stock no. 008377)) mice were bred at the animal facility of the Department of Veterinary Medicine, Robert-von-Ostertag Haus, Freie Universität Berlin, Germany, from a line originally developed in the S. Akira laboratory [62]. *Tlr2/4*^−*/*−^ mice were bred at the Charité animal facility, Berlin, Germany. Animals were maintained and handled in accordance with the German Animal Protection Law and approved by the Regional Office for Health and Social Services in Berlin (Landesamt für Gesundheit und Soziales – LAGeSo, Berlin, Germany).

Oli-neu cells were generously provided by Dr. J. Trotter (Institute of Molecular Biology, Johannes Gutenberg-University, Mainz, Germany [11]) and were cultured in Dulbecco’s Modified Eagle Medium (DMEM) supplemented with 10% fetal bovine serum (FBS) and 1% penicillin/streptomycin (DMEM complete; all obtained from Invitrogen, Darmstadt, Germany).

Mouse glioma (GL) 261 cells (Charles River Laboratory, Wilmington, MA, USA) were lentivirally transduced for fluorescence labeling. Briefly, mCherry gene was cloned into a lentiviral vector on a pRRL backbone (https://www.addgene.org/12252/), downstream of the MP71 promoter. After production of viral particles, GL261 cells were transduced at an MOI (multiplicity of infection) of 1 for 24 h using Roswell Park Memorial Institute (RPMI) medium supplemented with 10% fetal calf serum (FCS), 2% glutamine, 1% penicillin/streptomycin, and 0.4 mg/ml polybrene (all obtained from Merck, Darmstadt, Germany). Transduction was stopped by adding fresh medium. MCherry-labeled GL261 cells were selected via FACS cell sorting and frozen until further use.

### Primary cell cultures of microglia, astrocytes, and neurons

Neonatal primary microglia cultures were prepared from cerebral cortex and midbrain of newborn male and female P0-P3 C57BL/6, *Tlr5*^−*/*−^, or *Tlr2/4*^−*/*−^ mice, as described previously [40, 55]. In brief, forebrain was freed of blood vessels and meninges. Cortical tissue was dissociated by a fire-polished pipette using 3 ml of 2.5% trypsin for 2 min and washed twice with phosphate-buffered saline (PBS). Mixed glial cells were cultured for 9-12 d in DMEM (Invitrogen, Darmstadt, Germany) supplemented with 10% FCS and 1% penicillin/streptomycin (Gibco, New York, USA), with medium change every third day. Microglial cells were separated from the underlying glial layer by gentle shaking of the flasks for 1 h at 37 °C in 5% CO_2_ humidified atmosphere on a shaker (100 rpm) and plated (for densities, see specific experimental approaches below). Resulting cell cultures usually contained > 95% microglia, as detected by isolectin b4 (IB4) staining (for details see below). Cells were maintained at 37 °C in 5% CO_2_ humidified atmosphere.

Microglia from adult mice were prepared as described previously [54]. In short, brains derived from P49-P56 male mice were freed of blood vessels and meninges, mechanically dissociated, and digested with 2.5% trypsin and 1 mg/ml DNase (Roche Diagnostics, Mannheim, Germany). After further dissociation, cells were plated on a confluent monolayer of P0-P2 astrocytes in 75-cm^2^-flasks. The feeding astrocytic layer was depleted of neonatal microglia using clodronate (200 µg/ml) before plating the microglial cells (for densities, see specific experimental set-ups below). Mixed adult and neonatal glial cultures were maintained in fresh DMEM, supplemented as described above, and medium was changed every third day, followed by addition of 33% L292-conditioned medium at day 7. After a further week, cells were shaken off and used for experiments within 24-48 h.

Cultures of cortical neurons were generated from forebrains of E17 mice, as described previously [40]. In brief, brains were separated from blood vessels, meninges, and cerebellum. Cortical tissue was treated with 500 µl of 2.5% trypsin (Gibco, New York, USA) for 20 min at 37 °C. Trypsin activity was stopped by 4 ml FBS (Invitrogen, Darmstadt, Germany). Thereafter, cells were washed with PBS and incubated with 100 µl DNase (1 mg/ml; Roche Diagnostics, Mannheim, Germany) for 1 min. Subsequently, cells were washed, centrifuged, and seeded on poly-d-lysine-coated (Sigma-Aldrich, St. Louis, USA) glass coverslips with a diameter of 12 mm, at a density of 500,000 cells/well of a 24-well-plate, unless stated otherwise, with Neurobasal Medium (Gibco) supplemented with 1% L-Glutamin, 1% penicillin/streptomycin, and 2% B27 supplement (all obtained from Gibco, New York, USA). Cells were maintained at 37 °C in 5% CO_2_ humidified atmosphere. On the following day, half of media was replaced, and cells were incubated for additional 48 h before starting experiments.

Primary astrocytes were isolated, as previously described [39]. In detail, mixed glial cultures were prepared from mouse brains, as described above (see neonatal primary microglia). After separation of microglial cells from the underlying glial layer by gentle shaking of the flasks for 1 h at 37 °C on a shaker (100 rpm), cells were trypsinized (2.5%), and subsequently plated at a density of 80,000 cells/well of a 24-well-plate for immunocytochemical analysis.

For microglia/neuron co-culture experiments half of the medium of 500,000 cultured neurons was replaced by DMEM containing 62,500 microglia (ratio 8:1) at day 3 after plating. On the following day, cultures were used for experiments.

### Multiplex immune assay

Detection of TNF-α, IL-6, IL-10, IL-1β, GRO-α (CXCL1), macrophage inflammatory protein 2 (MIP-2 or CXCL2) and RANTES (CCL5) in 50 μl supernatant collected from microglial cell cultures (density of 30,000/200 μl medium/well of a 96-well-plate) stimulated over time with flagellin was performed using the ProcartPlex mouse Multiplex Immunoassay Mix & Match (Affymetrix eBioscience, Vienna, Austria) according to the manufacturer’s manual. Briefly, the magnetic bead-based assay enables the simultaneous detection and quantification of multiple proteins in a single sample. Prior to incubation, samples were vortexed followed by centrifugation to remove particles. Proteins were detected using Luminex 200. Analysis was performed using Bioplex System Software 4.0 (Bio-Rad, Hercules, CA, USA).

### Nitric oxide release assay

The amount of nitric oxide (NO) was determined using the colorimetric Griess reaction (Sigma-Aldrich, St. Louis, MO, USA), as previously described [41]. In detail, primary neonatal cultured microglia were seeded in a 96-well-plate at a density of 30,000/well. Cells were incubated in DMEM complete (see above) for 24 h in a total volume of 200 µl/well. Flagellin was added at a concentration of 100 ng/ml. 100 ng/ml LPS was used as positive control. After 24 h, 100 µl of supernatant were transferred to a fresh 96-well-plate. Solution A (100 mg naphthylethylene in 50 ml aqua dest.) and solution B (1 g sulfanilamide and 6 ml 85% H_3_PO_4_ in 44 ml aqua dest.) were mixed at the ratio of 1:1 shortly before use. Subsequently, the mixed solution was added to the wells containing the microglial supernatant at 1:1 ratio. A nitrite standard curve was prepared. Absorbance was measured at 550 nm.

### Agarose spot assay

Agarose spot assay for chemotactic invasion was performed, as previously described [63]. In summary, 0.1 g of low-melting point agarose (Promega, Madison, WI, USA) was diluted in 20 ml of PBS originating a 0.5% agarose solution. Subsequently, the solution was heated until boiling and then cooled down to 40 °C. Afterwards, 90 µl of agarose solution were mixed with 10 µl of PBS with or without flagellin at indicated doses in a 0.5 ml Eppendorf tube. Ten µl of the mixed solution were plated onto 35- mm-glass-bottomed dishes (Mattek Corporation, Ahland, MA, USA) and were cooled down for 10 min at 4 °C. Four spots were pipetted in one dish, of which two contained PBS only and two contained PBS with flagellin. 500,000 microglial cells were plated onto the dish in 2 ml DMEM complete (see above) and incubated at 37 °C in 5% CO_2_ humidified atmosphere for 3 h. Subsequently, cells inside the spot were quantified by microscopy. To assess microglial motility versus chemotaxis, flagellin was additionally applied to the medium. For chemotaxis assays, flagellin was only present within the spot. Values are expressed as the average obtained from 6 dishes. When inhibitors (LY294002, 25 or 50 μM; Wortmannin, 0.1 or 1 μM; rapamycin, 100 μM; Akt inhibitor IV; 1 or 10 μM) were added, cells were pre-treated for 30 min before plated onto the dish.

### Boyden chamber assay

Flagellin-induced chemotaxis was tested using a 48-well-microchemotaxis Boyden chamber (Neuroprobe, Bethesda, MD, USA). Upper and lower wells were separated by a polycarbonate filter (8 μm pore size; Poretics, Livermore, CA, USA). 20,000-40,000 microglial cells in 50 μl of serum-free DMEM were added to the upper compartment, while lower wells contained flagellin in serum-free DMEM. Serum-free DMEM alone was used as control. For signaling inhibition experiments, cells were pre-treated with PI3K inhibitors (LY294002; 25 or 50 μM; Wortmannin; 0.1 or 1 μM), mTOR inhibitor (rapamycin; 10 or 100 μM), and with anti-mTLR5 antibody (1 μg/ml) for 30 min on ice before added to the upper compartment. The chamber was incubated at 37 °C and 5% CO_2_ for 6 h. Cells remaining on the upper surface of the membrane were removed by wiping, while cells in the lower compartment were fixed in methanol for 10 min and subjected to Diff-Quik stain (Medion Grifols Diagnostics AG, Duedingen, Switzerland). Rate of microglial migration was calculated counting cells in 4 random fields of each well using a 20 × bright-field objective. For each condition, 2-4 fields in 4-8 wells were analyzed. Under control conditions, ~ 40-80 cells were present per field, and numbers of cells in each field were normalized to the average derived from control condition (set to 100%).

### Flow cytometry-based phagocytosis assay

After isolation of microglia as described above, cells were resuspended in DMEM (Thermo Fisher Scientific, Waltham, MA, USA) supplemented with 10% FCS and 2 ml l-glutamine at 37 °C. Microglial cells were seeded onto primary cultured neonatal astrocytes overnight, as described previously [46]. Fluoresbrite Carboxylate Micropheres (BrightBlue, 4.5 μm) were coated with 5% FCS for 30 min at room temperature on a shaker at 1000 rpm. Beads were resuspended in Hank’s balanced salt solution at a final concentration of 2 x 10^6^ beads per ml. Microglia-astrocyte co-cultures were washed once with Hank’s balanced salt solution before 1 ml bead solution was applied. Cells were incubated with beads for 30 min at 37 °C in 5% CO_2_ humidified atmosphere. Afterwards, they were washed in fluorescence-activated cell sorting buffer. Cells were stained with CD11b antibody (1:50; eBioscience, San Diego, CA, USA) for 15 min at 4 °C. After staining, cells were washed in fluorescence-activated cell sorting buffer and pulled down at 500 g for 5 min. Cells were resuspended in propidium iodide solution (ratio of 1:1000 in fluorescence-activated cell sorting buffer) to stain dead cells. Stained cells were transferred to a BD LSRFortessa Flow cytometer (BD Bioscience, Heidelberg, Germany). The phagocytic index implements the total number of cells that phagocytosed, and the number of beads taken up by a given cell. Data were analyzed using FlowJo v10 software (Tree Star, Ashland, OR, USA).

### Toxicity assay in vitro

For toxicity studies, indicated amounts of flagellin and other reagents were added to neuron/microglia co-cultures (ratio 8:1; 500,000 neurons and 62,500 microglial cells per well of a 24-well-plate) or isolated neurons (500,000 neurons per well of a 24-well-plate) for various time periods, as indicated. Control cultures remained unstimulated. LPS (100 ng/ml) served as positive control for microglia-mediated neurodegeneration in co-cultures. Loxoribine (1 mM) served as positive control for TLR7-mediated apoptosis of neurons. For each condition, experiments were performed in duplicates. NeuN- and TUNEL-positive cells (in situ Cell Death Detection Kit, TMR red or Fluorescein, Roche Diagnostics, Mannheim, Germany) were counted analyzing 5 fields per coverslip. Control condition was set to 100%. Numbers of NeuN- and TUNEL-positive cells observed for each condition were compared with control.

### Intrathecal injection

Intrathecal injection of 6-8-week-old male C57BL/6 mice with 1 μg flagellin in a volume of 40 μl PBS was performed as described previously [25, 36]. PBS alone served as control. After transcardial perfusion with 4% paraformaldehyde, brains were removed and cryoprotected in 30% sucrose. Neuronal survival and microglial numbers were analyzed by blinded quantification of cortical NeuN-/active caspase-3-positive cells and Iba1-positive cells, respectively, in 6 fields (magnification x60) of 5 representative sections of each brain (see below).

### Glioma ex vivo model

Organotypic brain slice preparation was performed as previously described [45]. Brains from P14-P16 C57BL/6 and *Tlr5*^−*/*−^ mice were placed in ice-cold PBS. Cerebellum and olfactory bulb were removed and the forebrain cut in the coronal plane into 250-μm-sections using a vibratome (Microm HM650V, Thermo Scientific, Waltham, MA, USA). Brain slices were transferred onto 0.4 μm-polycarbonate membranes of a transwell insert (Falcon model 3090, Becton–Dickinson, Franklin Lakes, NJ, USA), which was inserted into a 6-well-plate (Falcon model 3502, Becton–Dickinson, Franklin Lakes, NJ, USA). Brain slices were incubated in 1 ml of culture medium per well containing DMEM supplemented with 10% heat-inactivated FBS (Gibco, Thermo Fisher Scientific, Waltham, MA, USA), 0.2 mM glutamine, and 1% penicillin/streptomycin). After overnight equilibration, slices were incubated with medium containing 25% FBS (Atlanta Biological, Norcross, GA, USA), 50 mM sodium bicarbonate, 2% glutamine, 25% Hank’s balanced salt solution, 1 μg/ml insulin, 5 mM Tris (all from Life Technologies, Carlsbad, CA, USA), 2.46 mg/ml glucose (Braun, Melsungen, Germany), 0.8 mg/ml vitamin C, 100 U/ml penicillin, and 100 μg/ml streptomycin (all from Sigma-Aldrich, St. Louis, MO, USA) in DMEM (Gibco, Thermo Fisher Scientific, Waltham, MA, USA). Slices were cultured for 5 d with medium change every 2 d. For flagellin stimulation experiments, flagellin (100 ng/ml) was added to the medium on day 1 and 3 after slicing.

The day after sectioning, 5000 cultured mCherry GL261 glioma cells in a volume of 0.1 μl were injected into the slices using a microsyringe mounted to a micromanipulator. An injection canal was formed that reached 150 μm deep into the 250-μm-thick slice. The needle was then retracted by 50 μm, thereby forming an injection cavity of about 50 μm depth. The tumor cell suspension was slowly inoculated into the cavity, and the syringe was pulled out. To ensure identical experimental conditions, gliomas were always inoculated in the striatal area of both hemispheres.

On day 4 after injection organotypic brain slices were fixed with 4% PFA for 1 h, washed with PBS and stained with Hoechst (1:4750) before being washed again and mounted. Tumor volumes were determined by confocal microscopy (Leica TCS SPE, Leica Microsystems, Wetzlar, Germany). Images were acquired with a 20x oil immersion objective using a z-stack with a 2-μm-step-size interval and tile scan mode. Tumor volume was assessed using Imaris 9.2 (Bitplane, Zürich, Switzerland). Tumor volumes were 3D-rendered by application of 1 μm object detail, 15-threshold background, and 1000 tridimensional pixels (voxels). The surface objects obtained were unified in one single object, and values for volume mean were extracted.

### Immunocytochemistry, immunohistochemistry, and TUNEL assay

Immunostaining was performed as described previously [41]. In detail, representative brain sections (level 1: interaural 6.60 mm; level 2: 5.34 mm; level 3: 3.94 mm; level 4: 1.86 mm; level 5: −0.08 mm) or cell cultures (microglia at a density of 80,000/well, astrocytes at a density of 80,000/well, neurons at a density of 300,000/well; all cell types were seeded on 12-mm-glass cover slips in a 24-well-plate) were fixed with 4% PFA (200 μl), washed 3x with 200 μl PBS, and treated with blocking solution (5% normal goat serum (NGS) for brain sections or 2% NGS for cell cultures; 0.2% TritonX-100) for 1 h. The following primary antibodies were used: TLR5 (clone 19D759.2, ab13876, Abcam, Cambridge, UK, 1:200), NeuN (ABN 78, Sigma-Aldrich, St. Louis, MO, USA, 1:500), active caspase-3 (#9661, Asp175, Cell Signaling Technology, 1:500), S100β (ab227914; Abcam, Cambridge, UK, 1:250) and Iba1 (#019-19741, Wako, 1:500). Isolectin b 4 (IB4) was obtained from Invitrogen (Carlsbad, CA, USA). Cells and brain sections were incubated with the respective antibody overnight at 4 °C. Subsequently, they were washed 3x with PBS incubated with the relevant secondary antibody (# A-11001, Alexa Fluor 488 goat anti-mouse IgG; # A-11004, Alexa Fluor 568 goat anti-mouse IgG; # A-11008, Alexa Fluor 488 goat anti-rabbit IgG; # A-11011, Alexa Fluor 568 goat anti-rabbit IgG; all used in 1:500 dilution and purchased from Invitrogen, Eugene, OR, USA) for 1 h at room temperature. Finally, nuclei were stained with 4′,6-diamidino-2-phenylindole (DAPI) solution (1:10,000, D9542, Sigma-Aldrich, St. Louis, MO, USA) for 1 min and washed 3x in PBS. Coverslips were attached onto glass slides using 1 drop of ImmuMount (#9990402; Thermo Scientific, Waltham, MA, USA)/coverslip.

Numbers of neurons and microglia in the cerebral cortex within brain sections were assessed by blinded quantification of NeuN- and Iba1-positive cells in 3 fields per hemisphere (x60 magnification) at level 4 of the 5 representative sections of each brain (see above). For analysis of apoptosis, brain sections were stained by active caspase-3 and DAPI and quantified as described for NeuN- and Iba1-stained cells above. The mean was calculated, which is expressed as NeuN-positive, Iba1-positive, or active caspase-3-positive cells per mm^2^. Each group is displayed with the median. An Olympus BX51 microscope and a Leica TCS SL confocal laser-scanning microscope were used.

### Western blot

Western blot analysis was performed using whole lysates of 10^6^ cultured microglia per dish. Before collection, cells were washed with cold PBS. Radioimmunoprecipitation assay (RIPA) buffer was used as lysis buffer containing 1% NP-40, 0.5% sodium deoxycholate, and 0.1% SDS dissolved in 1% TBS buffer. Protease inhibitor cocktail (Roche, Grenzach-Wyhlen, Germany) and phosphatase inhibitor mixture (Sigma-Aldrich, Darmstadt, Germany) were added to RIPA buffer before cell lysis. Cells were homogenized by a syringe needle, followed by centrifugation at 15,000 rpm for 10 min to remove insoluble cell debris. Protein concentration was measured by BCA protein assay kit (Thermo Fisher Scientific, Waltham, MA, USA), and a total of 20 μg protein was separated by 10% SDS-PAGE and subsequently electrophoretically transferred onto polyvinylidene difluoride (PVDF) membranes (Bio-Rad Laboratories, Hercules, CA, USA). After protein transfer, PVDF membranes were blocked with Tris-buffered saline containing 5% bovine serum albumin (BSA) for 1 h at room temperature. Membranes were then incubated at 4 °C overnight with antibodies against phospho-AKT (S-473) and Akt purchased from Cell Signaling Technology (Danvers, MA, USA). Membranes were washed and incubated with anti-rabbit IgG secondary antibody (Cell Signaling Technology, Danvers, MA, USA) at room temperature for 2 h. Protein signal transferred onto the PVDF membrane was visualized by ECL Prime Western Blotting Detection Reagent (GE Healthcare, Buckinghamshire, UK) using the Chemi Doc XRS system (Bio-Rad Laboratories, Hercules, CA, USA). Quantification of signals was assessed using Image J software.

### Quantitative real-time PCR

Primary neonatal microglia, adult microglia, astrocytes, neurons, or Oli-neu cells were seeded at a density of 10^6^ cells per dish in a 6-well-plate. After 24 h, cells were stimulated with flagellin (100 ng/ml) for 24 h. Subsequently, cell lysates were collected and RNA was extracted, followed by cDNA synthesis and quantitative PCR expression analysis. Primers directed against TLR5 and TNF-α were purchased from QIAGEN (Hilden, Germany). For details of the qPCR cycle program used please refer to the *Thermo Scientific Maxima SYBR Green/ROX qPCR Master Mix* manual.

### Statistics

Data are expressed as mean ± SEM or ± SD. Statistical differences between selected groups were determined using Dunnett’s or Tukey’s multiple comparison test after one-way ANOVA, Kruskal–Wallis test followed by Dunn’s multiple comparison post hoc test, or Student’s *t* test, as indicated. Statistical differences were considered significant when *P *< 0.05.

## Results

### In the CNS, TLR5 is primarily expressed in microglia, and its activation triggers PDK1 and Akt phosphorylation in a PI3K-dependent fashion

**Fig. 1:**
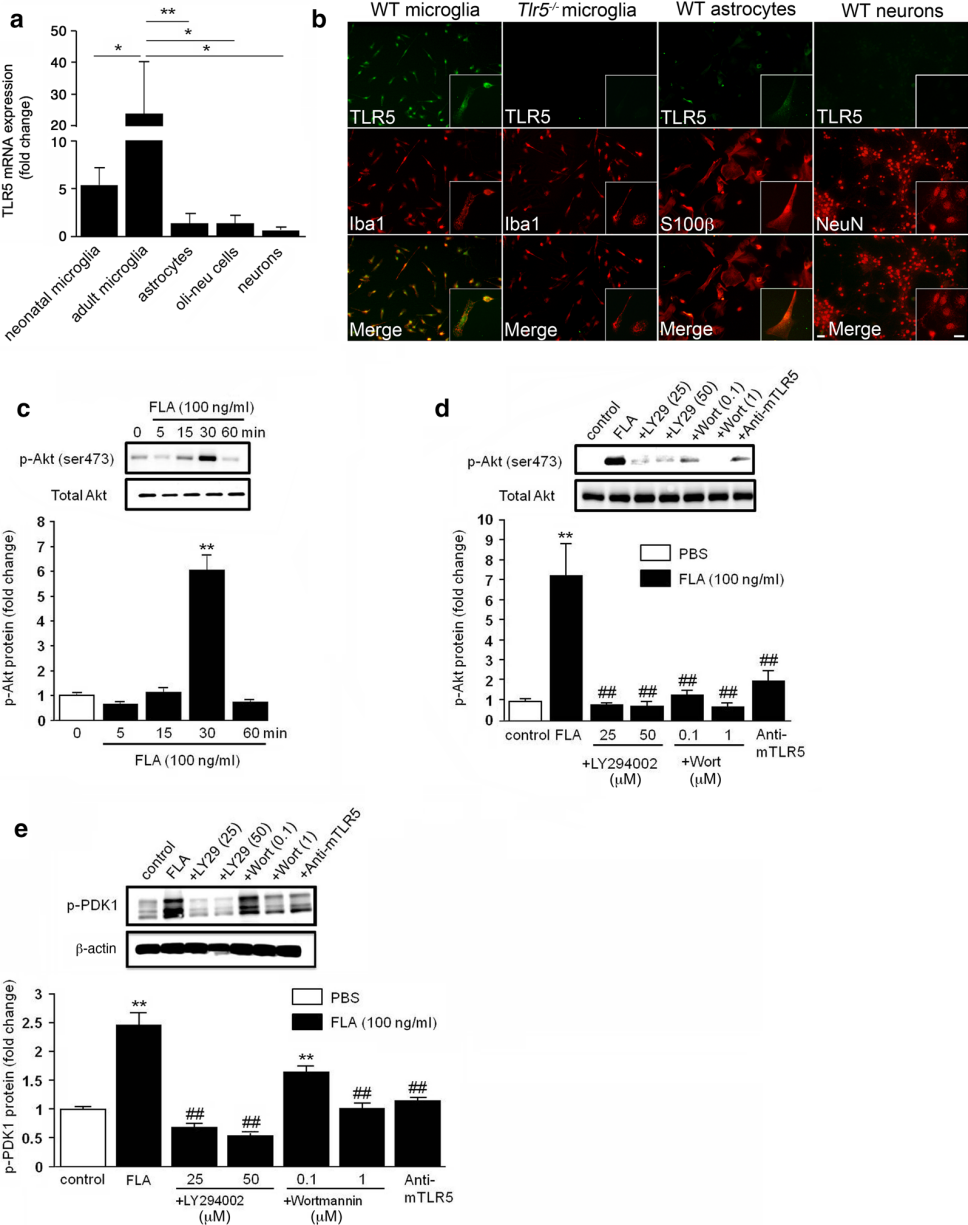
TLR5 is constitutively expressed in microglia, and its activation triggers PDK1 and Akt phosphorylation in a PI3K-dependent manner. **a** Relative TLR5 expression levels were assessed in primary neonatal microglia, primary adult microglia, primary astrocytes, and primary cortical neurons isolated from C57BL/6 mice, as well as Oli-neu cells by quantitative RT-PCR (fold-change compared to neurons). TATA sequence binding protein (TBP) was used as housekeeping control (*n* = 3). Results are represented as mean ± SD. Data were analyzed by one-way ANOVA followed by Newman-Keuls test. *P < 0.05; **P < 0.01. **b** Cultured neonatal microglia, astrocytes, and neurons from C57BL/6 (WT) mice, as well as microglia from *Tlr5*^−*/*−^ mice were stained with antibody directed against TLR5 and co-stained with Iba1, S100β, or NeuN antibody serving as microglial, astrocyte, and neuronal marker, respectively. Scale bar, 10 μm. **c** Western blot analysis of microglial lysates using antibodies against p-Akt and total Akt after incubation of microglia with 100 ng/ml flagellin (FLA) for 0, 5, 15, 30, or 60 min (*n* = 3). Representative blots are shown in the upper panel. The graph depicts the average intensity ratio of the bands compared to control (lower panel). Data are expressed as mean ± SEM and were analyzed by one-way ANOVA followed by Dunnett’s post hoc test. **P < 0.01 vs. control. **d** Western blot analysis of FLA-mediated Akt phosphorylation after treatment of microglia with LY294002 (25 and 50 μM), Wortmannin (0.1 and 1 μM), and anti-mTLR5-IgG (1 μg/ml) (upper panel, *n* = 3). The graph shows the average intensity ratio of the bands compared to control (lower panel). **e** Western blot analysis of microglial lysates with antibodies against p-PDK1 and β-actin after incubation of microglia with 100 ng/ml FLA, FLA plus LY294002 (25 and 50 μM), FLA plus Wortmannin (0.1 and 1 μM), and FLA plus anti-mTLR5-IgG (1 μg/ml) (upper panel, *n* = 3). The graph depicts average intensity ratio of the bands compared to control (lower panel). Data are expressed as mean ± SEM and were analyzed by one-way ANOVA followed by Tukey’s post hoc test. **P < 0.01 vs. control; ^##^*P* < 0.01 vs. FLA. **d**, **e** DMSO-containing DMEM served as control, while FLA was solved in DMSO-containing DMEM

TLR5 was previously reported being expressed in microglia and dorsal root ganglion neurons [52, 64]. We compared expression levels of TLR5 in murine CNS glial cells and neurons by quantitative RT-PCR. Among the different CNS cell types (for images of cultured primary microglia, neurons, and astrocytes, please refer to Additional file 1), microglia expressed the highest levels of TLR5, of which adult microglia expressed higher TLR5 expression levels than neonatal microglia. Compared to microglia, astrocytes and Oli-neu cells, a mouse oligodendroglial precursor line, expressed low levels of TLR5, while this receptor was barely detectable in primary cortical neurons (Fig. 1a). TLR5 protein was readily detectable in cultured neonatal microglia, but also in astrocytes, although to a much lesser extent, by immunocytochemistry. In contrast, TLR5 protein expression was not detected in cortical neurons (Fig. 1b). Specificity of the antibody to TLR5 was confirmed by analyzing microglia from *Tlr5*^−*/*−^ mice (Fig. 1b).

Flagellin-induced TLR5 activation was previously shown to induce PI3K, as evidenced by Akt phosphorylation, a downstream effector of PI3K and 3-phosphoinositide-dependent kinase-1 (PDK1), thereby mediating regulation of proinflammatory gene expression in epithelial cells [51, 67]. To investigate this signaling pathway in microglia, we exposed neonatal microglia to flagellin and subsequently assessed their p-Akt expression by western blot. In response to flagellin, p-Akt expression was significantly increased after 30 min and decreased to control level at 60 min (Fig. 1c). Flagellin-induced Akt phosphorylation was abolished by the PI3K inhibitors LY294002 and Wortmannin (Fig. 1d). Furthermore, flagellin-induced Akt phosphorylation was dependent on functional TLR5, as TLR5-neutralizing antibody significantly diminished this response (Fig. 1d). Next, flagellin-treated microglia were analyzed for phosphorylated PDK1 (p-PDK1) expression. Western blot analysis revealed an increase in p-PDK1 expression in response to flagellin after 30 min, and LY294002 and wortmannin significantly inhibited these effects (Fig. 1e). Employment of the TLR5-neutralizing antibody reduced levels of p-PDK1, thereby confirming TLR5 as the signaling receptor in flagellin-induced PDK1 phosphorylation (Fig. 1e).

### Activation of TLR5 and subsequent PI3K/Akt/mTORC1 signaling in microglia results in the release of distinct inflammatory molecules

**Fig. 2:**
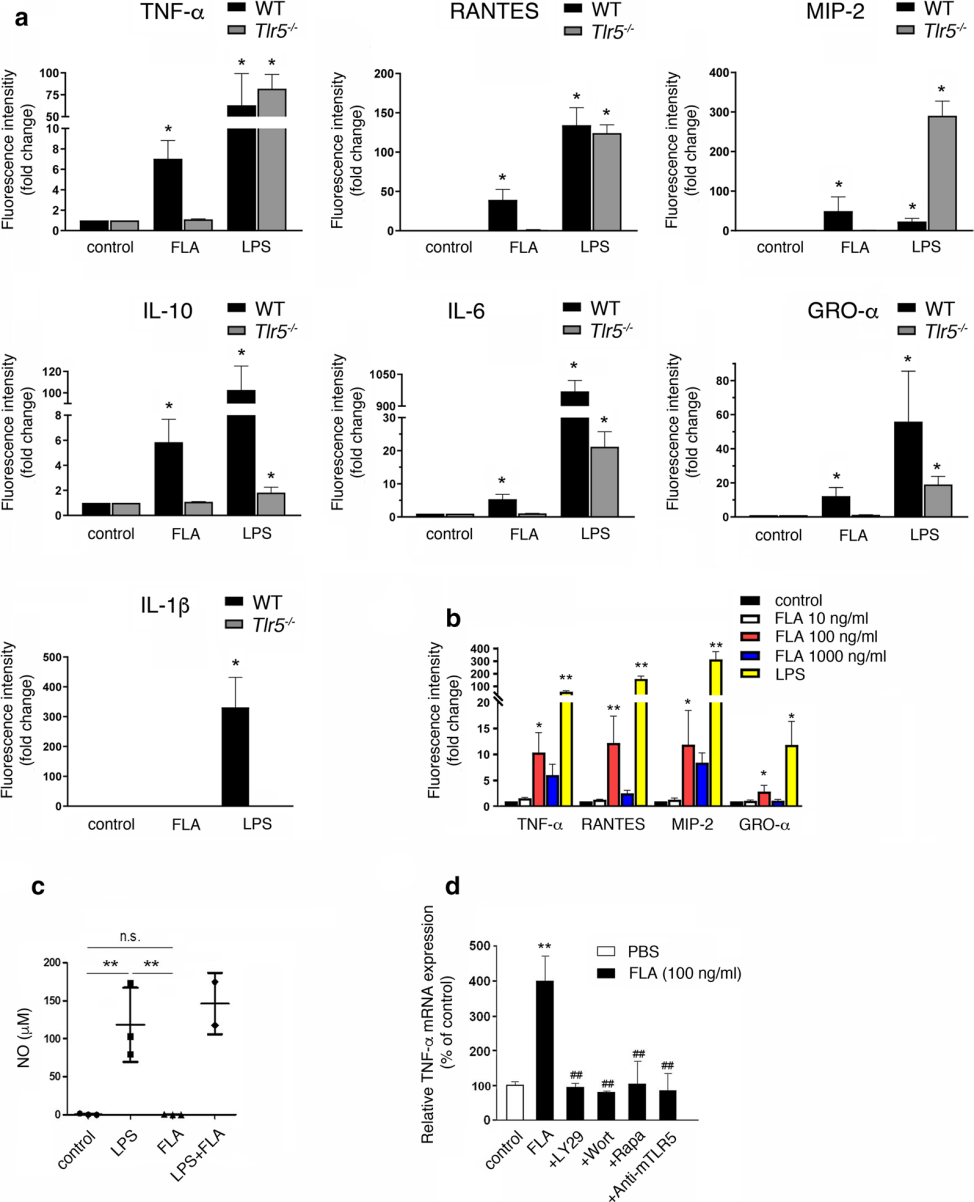
Flagellin induces the release of cytokines and chemokines from microglia through TLR5 signaling. **a** Multiplex immunoassay was used to detect cytokines/chemokines, as indicated, in supernatants of cultured neonatal microglia from C57BL/6 (WT) and *Tlr5*^−*/*−^ mice in response to 100 ng/ml flagellin (FLA) after 24 h. Unstimulated cells served as negative control, while LPS (100 ng/ml) was used as positive control (*n* = 3). **b** Supernatants of cultured neonatal WT microglia stimulated with 10 ng/ml, 100 ng/ml, 1000 ng/ml FLA, or 100 ng/ml LPS were analyzed for indicated cytokines/chemokines after 24 h by multiplex immunoassay. Unstimulated cells served as control (*n* = 3). Results are expressed as mean ± SEM. Data were analyzed by Kruskal–Wallis test followed by Dunn’s post hoc test. *P < 0.05; **P < 0.01 vs. control. **c** Cultured neonatal WT microglia were stimulated with 100 ng/ml FLA and/or 100 ng/ml LPS for 24 h, and supernatants were analyzed for NO using Griess assay (*n* = 3). Results are expressed as mean ± SEM. Data were analyzed by Kruskal–Wallis test followed by Dunn’s post hoc test. **P < 0.01 vs. control; n.s., not significant. **d** WT microglia were incubated with 100 ng/ml FLA alone or in combination with LY294002 (50 μM), Wortmannin (1 μM), rapamycin (100 μM), or anti-mTLR5-IgG (1 μg/ml) for 6 h. DMSO-containing DMEM served as control, while FLA was solved in DMSO-containing DMEM. Subsequently, quantitative RT-PCR using primers against TNF-α was performed. TBP served as housekeeping gene (*n* = 5). Results are expressed as normalized to control and are represented as mean ± SD. Data were analyzed by one-way ANOVA followed by Tukey’s post hoc test. **P < 0.01 vs. control; ^##^*P* < 0.01 vs. FLA

Activation of various TLRs results in the release of inflammatory molecules including cytokines, chemokines, and nitric oxide (NO) from microglia [37]. However, the role of TLR5 in microglia in this context has not been explored. Thus, to determine the specific inflammatory response pattern induced by activation of microglial TLR5, we analyzed supernatants from neonatal wild-type (WT) and *Tlr5*^−*/*−^ microglia incubated with 100 ng/ml flagellin after 24 h by multiplex immunoassay. Specific cytokines and chemokines were selected for analysis based on their relevance in the context of neuroinflammation and neurodegenerative diseases, as well as on their suitability as microglial activation markers [23, 50, 58]. LPS as the established TLR4 ligand served as positive control. TNF-α, RANTES, MIP-2, IL-10, IL-6, and GRO-α were significantly released from WT microglia in response to flagellin, while IL-1β was not detected (Fig. 2a; for protein concentrations of detected cytokines/chemokines please refer to Additional file 2). Notably, LPS induced an enhanced MIP-2 response in *Tlr5*^−*/*−^ microglia compared to WT cells (Fig. 2a). Induction of all tested cytokines in flagellin-treated microglia required TLR5, as *Tlr5*^−*/*−^ microglia failed to respond to flagellin (Fig. 2a). To assess dose-dependent effects, microglia were incubated with increasing doses of flagellin up to 1000 ng/ml, and their supernatants were subsequently analyzed for selected cytokines by multiplex immunoassay. We observed a dose-dependent increase in TNF-α, RANTES, MIP-2, and GRO-α in response to 10 ng/ml and 100 ng/ml flagellin compared to control condition. However, microglia exposed to 1000 ng/ml flagellin released diminished amounts of these cytokines compared to microglia responding to 100 ng/ml flagellin (Fig. 2b). This cytokine reduction observed at 1000 ng/ml flagellin was not due to potential flagellin-induced toxicity towards microglia, as quantification of IB4-positive cells and TUNEL staining of flagellin-treated cells did not indicate cellular injury or loss, even when microglia were exposed to doses up to 5 μg/ml flagellin (Additional file 3). Induction of cytokines, as indicated above, in *Tlr2/4*^−*/*−^ microglia was similar to that of WT cells after exposure to flagellin, excluding the possibility of relevant contamination of recombinant flagellin with LPS or TLR2 ligands (data not shown) and confirming the results derived from *Tlr5*^−*/*−^ microglia with respect to receptor specificity. Further, in contrast to LPS, flagellin did not induce NO production in microglia, as assessed by Griess assay (Fig. 2c).

To determine whether flagellin-induced release of cytokines from microglia requires PI3K/Akt signaling, microglia were incubated with flagellin in the presence or absence of LY294002 or Wortmannin for 6 h, and mRNA expression levels of TNF-α-a representative cytokine produced in activated microglia, were determined by quantitative RT-PCR. As expected, PI3K inhibitor treatment inhibited TNF-α mRNA expression in microglia exposed to flagellin (Fig. 2d). Likewise, presence of the mammalian target of rapamycin complex 1 (mTORC1) inhibitor rapamycin in microglial cultures prevented TNF-α expression in these cells (Fig. 2d). Pre-treatment with the TLR5-neutralizing antibody also diminished flagellin-induced TNF-α mRNA expression in microglia, thereby confirming TLR5 as the responsible immune receptor in this context (Fig. 2d).

Taken together, flagellin induced the release of distinct cytokines from microglia depending on TLR5 and PI3K/Akt/mTORC1 signaling.

### Flagellin modulates microglial chemotaxis through TLR5 and PI3K/Akt/mTORC1 signaling

**Fig. 3:**
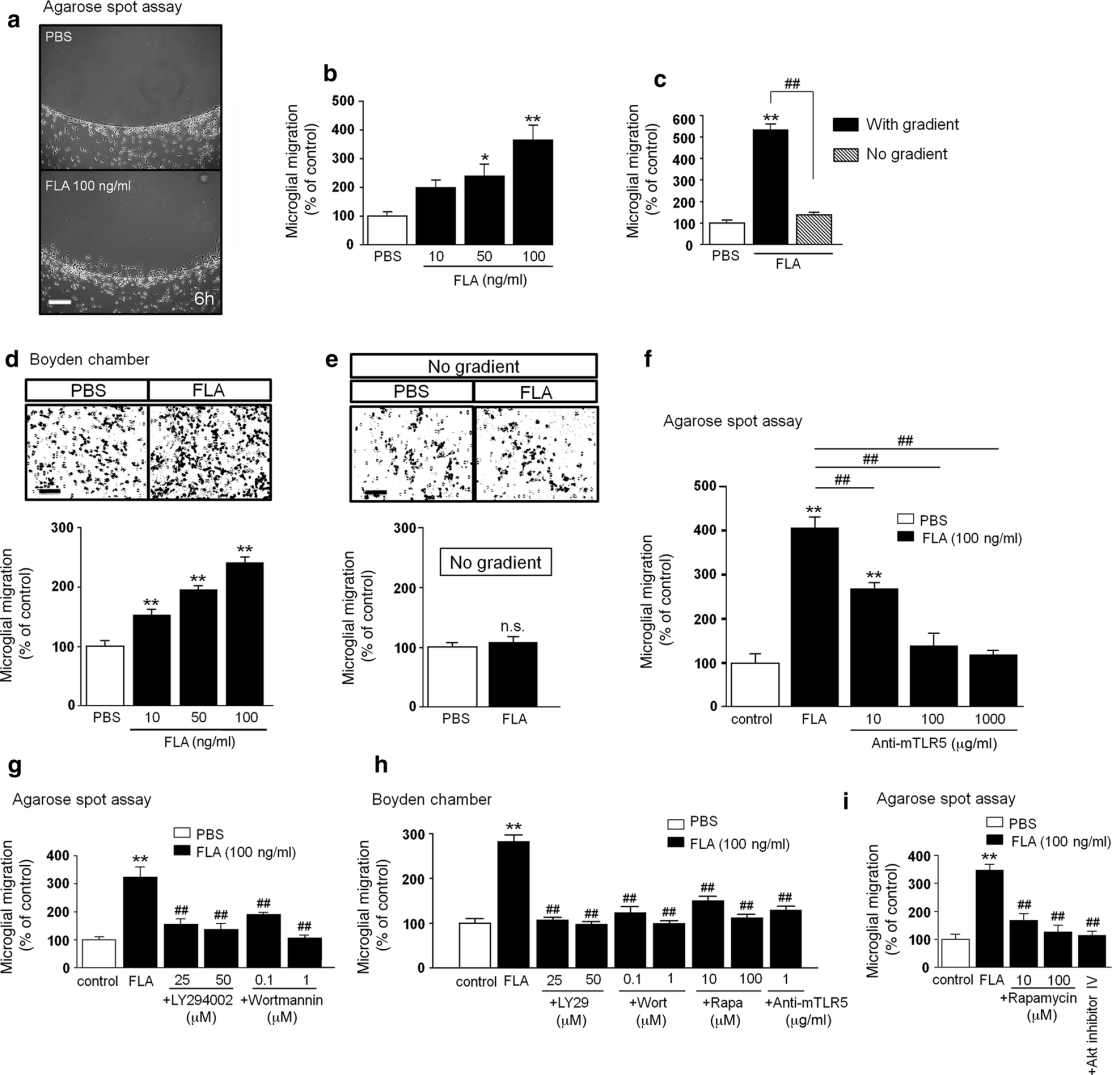
Flagellin triggers chemotaxis, but not random motility in microglia, through TLR5 and PI3K/PDK1/Akt/mTORC1 signaling. **a**–**c** Microglial migration in response to flagellin (FLA) was analyzed by agarose spot assay. **a** Images of FLA- and PBS (control)-treated C57BL/6 microglia. Scale bar, 100 μm. **b** Various FLA doses, as indicated, were added either to the spot alone (gradient/black, *n* = 8) or **c** to both the spot and the culture medium (no gradient/grey, *n* = 4). PBS was used as negative control. Microglial migration was quantified after 6 h of FLA incubation. Results are expressed as mean ± SEM. Data were analyzed by one-way ANOVA followed by Tukey’s post hoc test. *P < 0.05; ^**^P < 0.01 vs. control; ^##^*P* < 0.01. **d** Microglial migration in response to FLA was analyzed by Boyden chamber assay. Images of C57BL/6 microglia plated in the upper compartment and treated with 100 ng/ml FLA. PBS served as control. Scale bar, 100 μm (upper panel). Microglial migration in response to various FLA doses, as indicated, was quantified after 6 h (lower panel, *n* = 10). Results are expressed as mean ± SEM. Data were analyzed by one-way ANOVA followed by Dunnett’s post hoc test. ^**^P < 0.01 vs. control. **e** Quantification of microglia plated in both wells of the Boyden chamber lacking a gradient (*n* = 10). Results are expressed as mean ± SEM. Data were analyzed by one-way ANOVA followed by Dunnett’s post hoc test. n.s., not significant. **f** Agarose spot assay testing microglial migration, as described in (**a**, **b**) in the presence of various doses of mouse TLR5-neutralizing antibody (anti-mTLR5), as indicated (*n* = 4). Results are expressed as mean ± SEM. Data were analyzed by one-way ANOVA followed by Dunnett’s post hoc test. ^**^P < 0.01 vs. control; ^##^*P* < 0.01. **g** Agarose spot assay testing microglial migration, as described in (**a**, **b**) in the presence of LY294002 (25 and 50 μM) and Wortmannin (0.1 and 1 μM), *n* = 12. Results are expressed as mean ± SEM. Data were analyzed by one-way ANOVA followed by Tukey’s post hoc test. **P < 0.01 vs. control; ^##^*P* < 0.01 vs. FLA. **h** FLA-induced (100 ng/ml) microglial migration in the Boyden chamber in the presence of LY294002 (25 and 50 μM), Wortmannin (0.1 and 1 μM), rapamycin (10 and 100 μM), or anti-mTLR5-IgG (1 μg/ml), *n* = 12. Results are expressed as mean ± SEM. Data were analyzed by one-way ANOVA followed by Tukey’s post hoc test. ^**^P < 0.01 vs. control; ^##^*P* < 0.01 vs. FLA. (**i**) FLA-induced (100 ng/ml) microglial migration tested by agarose spot assay in the presence of rapamycin (10 and 100 μM) or Akt inhibitor IV (1 μM), *n* = 4. Results are expressed as mean ± SEM. Data were analyzed by one-way ANOVA followed by Tukey’s post hoc test. ^**^P < 0.01 vs. control; ^##^*P* < 0.01 vs. FLA. **g**–**i** DMSO-containing DMEM served as control, while FLA was solved in DMSO-containing DMEM

To test the effect of flagellin on microglial migration, we analyzed cell accumulation in an agarose spot containing either flagellin or PBS serving as control (Fig. 3a). Microglia that had migrated into the agarose spot after 6 h, were quantified. At a dose of 10 ng/ml flagellin, microglia did not significantly migrate, whereas the migration rate was significantly increased in response to 50 ng/ml and 100 ng/ml flagellin (Fig. 3b). To determine whether flagellin induces cell motility or directed migration, i.e. chemotaxis, 100 ng/ml flagellin was added either to the spot only, or to both spot and surrounding supernatant. Presence of flagellin increased microglial motility only in the presence of a gradient, as this effect was not observed when the gradient was absent (Fig. 3c). To confirm flagellin-induced chemotaxis, we tested microglia in a further migration assay, the Boyden chamber. Here, we observed similar effects as observed with the agarose spot assay. However, in the Boyden chamber, flagellin increased microglial migration at flagellin doses as low as 10 ng/ml (Fig. 3d). When flagellin was added to both top and bottom wells, thereby disseminating the gradient, no significant increase in microglial migration was detected (Fig. 3e). TLR5 served as the responsible receptor in flagellin-induced microglial chemotaxis, as pre-treatment with the TLR5-neutralizing antibody inhibited migration in a dose-dependent fashion (Fig. 3f).

We reported previously that TLR2 and TLR7 agonist-induced microglial migration is inhibited by treatment with the PI3K inhibitors LY294002 and wortmannin, suggesting that signaling through PI3K contributes to TLR-induced microglial migration [29]. To analyze the PI3K/Akt signaling pathway in flagellin-induced microglial chemotaxis, we again made use of the migration assays described above testing a dose of 100 ng/ml flagellin. In both agarose spot assay (Fig. 3g) and Boyden chamber (Fig. 3h) pre-treatment with LY294002 (at 25 and 50 μM) and Wortmannin (at 0.1 and 1 μM) for 30 min inhibited flagellin-induced microglial migration. To further characterize the signaling cascade through which PI3K regulates flagellin-induced migration, we included the established mTORC1 inhibitor rapamycin (at 10 and 100 μM) and Akt inhibitor IV (1 μM) in the migration analysis. Pretreatment with both inhibitors for 30 min inhibited flagellin-induced microglial motility, suggesting that this process is dependent on Akt and mTORC1 signaling (Fig. 3h, i). TLR5 as the responsible receptor in flagellin-induced microglial migration (see Fig. 3f) was confirmed using the Boyden chamber assay as pre-treatment with the TLR5-neutralizing antibody inhibited migration (Fig. 3h).

In summary, these results indicate that flagellin is a chemotactic signal for microglia mediated by TLR5 and PI3K/Akt/mTORC1 signaling.

### Microglial phagocytosis is enhanced in response to flagellin and dependent on TLR5 and PI3K/Akt/mTORC1 signaling

**Fig. 4:**
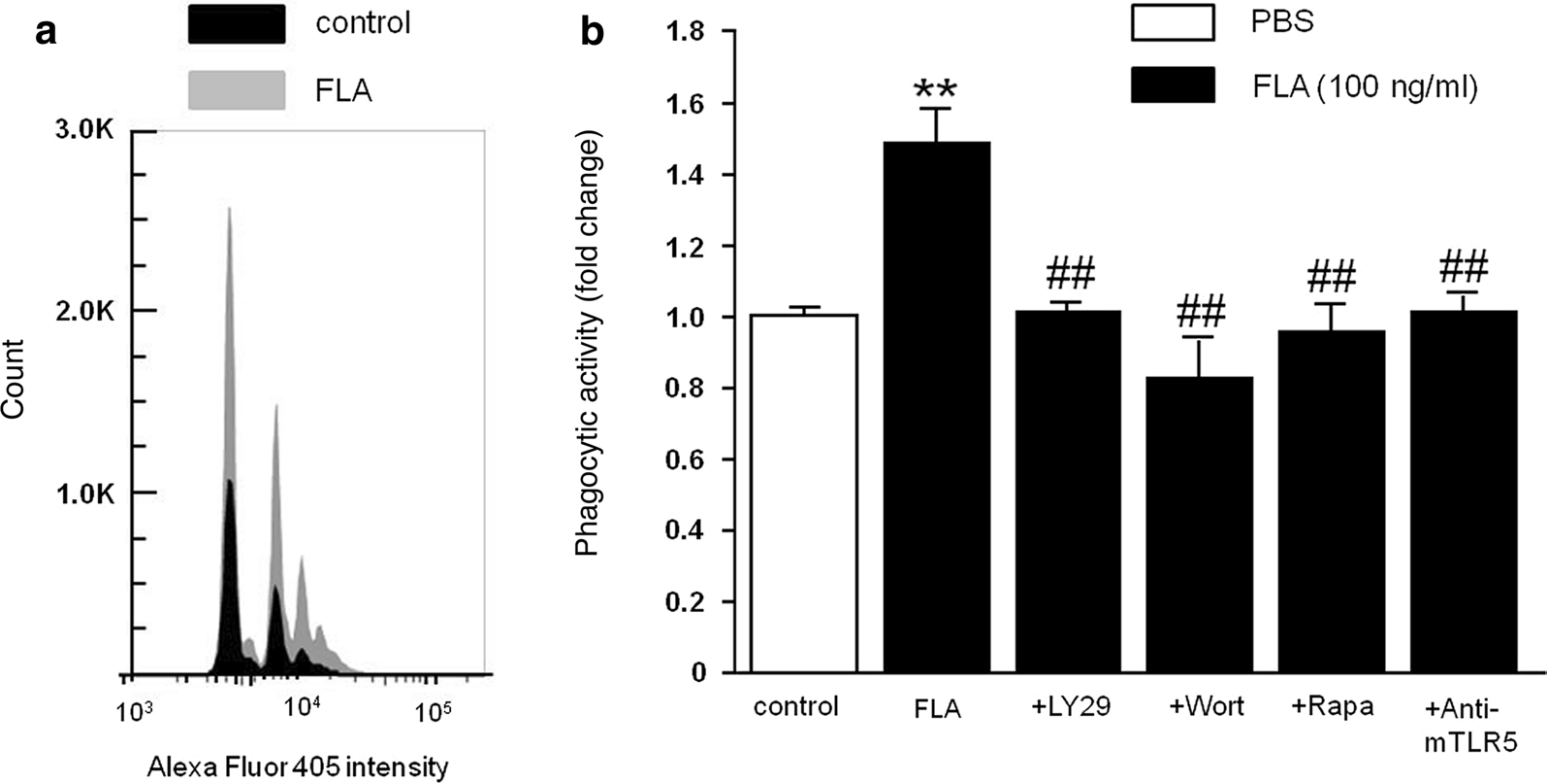
Flagellin-induced microglial phagocytosis is mediated by TLR5 and the PI3K/PDK1/Akt/mTOR pathway. **a** Using FACS-based phagocytosis assay incorporation of Alexa Fluor 405-labelled beads into microglia was quantified. The image shows representative histograms, in which peaks represent the number of microglia that phagocytosed none or one to four beads in the absence (control, black) and presence of flagellin (FLA, 100 ng/ml, gray). **b** The phagocytic index derived from the data shown in (**a**) was determined and normalized to control. In addition, FLA-treated microglia in the presence of LY294002 (50 μM), Wortmannin (1 μM), rapamycin (100 μM), or anti-mTLR5-IgG (1 μg/ml) were tested (*n* = 4). DMSO-containing DMEM served as control, while FLA was solved in DMSO-containing DMEM. Results are expressed as mean ± SEM. Data were analyzed by one-way ANOVA followed by Tukey’s post hoc test. ^**^P < 0.01 vs. control; ^##^*P* < 0.01 vs. FLA

To examine whether flagellin affects the phagocytic activity of microglia, we analyzed the uptake of fluorescent beads through flow cytometry. Fluorescent beads were added to microglia for 30 min, and subsequently, cells were isolated and stained with CD11b antibody. Exposure to flagellin for 30 min significantly increased phagocytic activity of microglia. The number of cells containing one, two, and more beads were significantly increased in flagellin-exposed cell cultures, while the number of cells lacking beads decreased (Fig. 4a, b). This effect was completely abolished in the presence of LY294002, Wortmannin, and rapamycin, indicating that PI3K/Akt/mTORC1 signaling was required in flagellin-induced microglial phagocytosis. Furthermore, increased phagocytic activity of microglia in response to flagellin was dependent on TLR5, as TLR5-neutralizing antibody abolished this effect (Fig. 4b).

### In contrast to TLR 2/4/7, TLR5 does not mediate microglia-glioma interaction

TLRs play an important role for the interaction of microglia with glioma cells promoting a pro-tumorigenic phenotype including TLR2, TLR4, and TLR7 signaling [18]. This has been substantiated by an ex vivo brain tumor model, in which mCherry-GL261 glioma cells were inoculated into brain slices [5, 12, 27]. Using this model, we explored whether TLR5 activation plays a role in glioma growth in brain slices derived from WT and *Tlr5*^−*/*−^ mice. Tumor sizes were compared after 4 d post-injection. No significant differences with respect to tumor volume between WT and *Tlr5*^−*/*−^ mice were detected (Additional file 4a). Likewise, when mCherry-GL261 glioma cells injected into WT brain slices were exposed to flagellin for 4 d, no significant impact on glioma growth was observed (Additional file 4b).

### Microglia exposed to flagellin contribute to neuronal apoptosis through TLR5

**Fig. 5:**
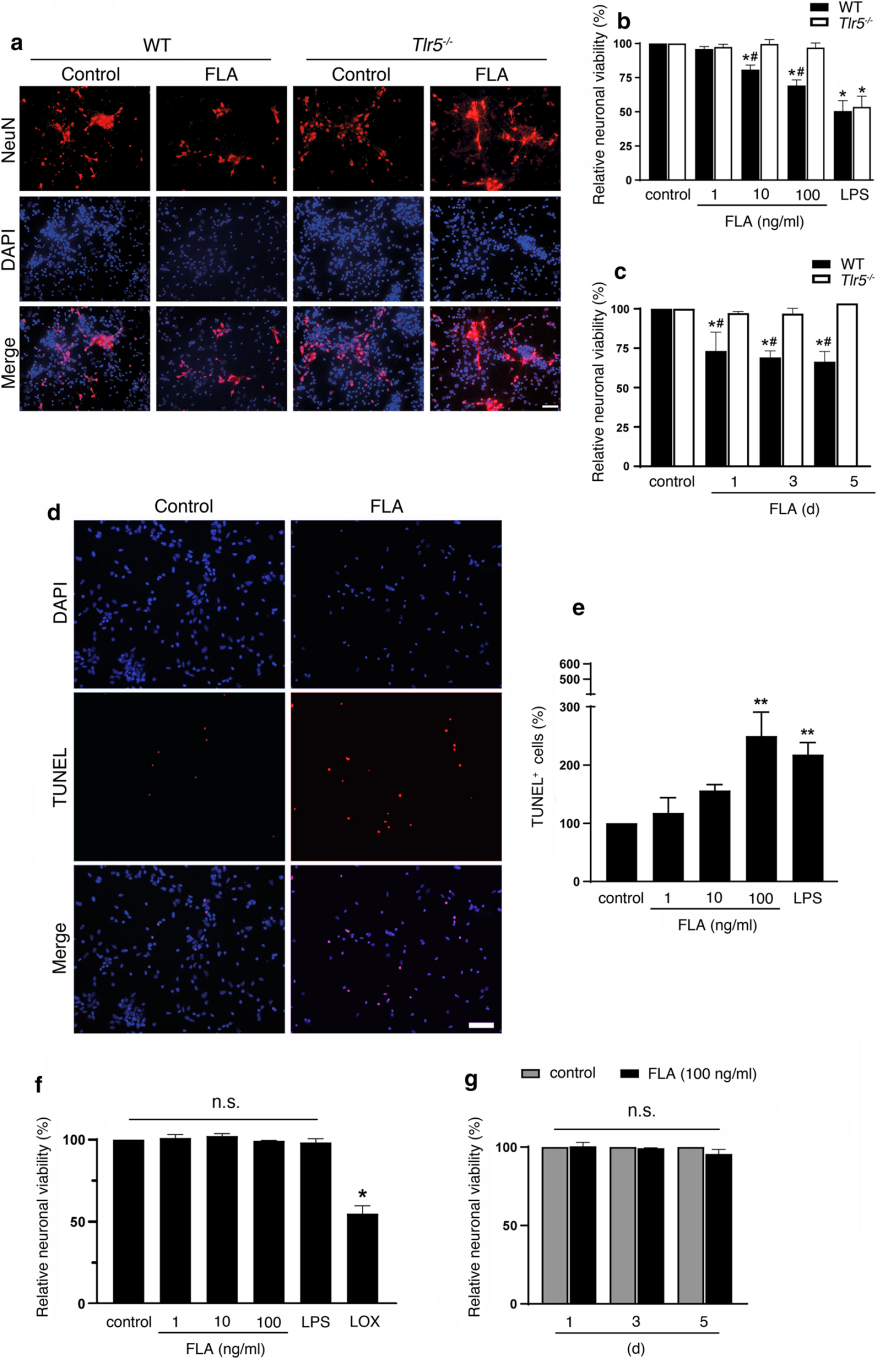
Flagellin induces neuronal cell death through TLR5 in vitro. **a** Co-cultures of C57BL/6 (WT) neurons and WT or *Tlr5*^−*/*−^ microglia were incubated with 100 ng/ml flagellin (FLA) for 72 h. Subsequently, cells were stained with NeuN antibody to mark neurons, and DAPI to label nuclei, and representative images are shown (scale bar, 50 μm; *n* = 3). **b**, **c** NeuN-positive cells in co-cultures treated with different FLA doses, as indicated, for 72 h (**b**), or with 100 ng/ml FLA for different time periods, as indicated (**c**), were quantified, and results were expressed as relative neuronal viability by setting the viability of control cells to 100%. 1 μg/ml LPS served as positive control, whereas unstimulated condition served as negative control (*n* = 3). **d** Images of TUNEL-positive and DAPI-labeled nuclei in unstimulated (control) and FLA-treated co-cultures containing WT microglia are shown (scale bar, 50 μm; *n* = 4). **e** Quantification of TUNEL-positive cells in co-cultures incubated with different FLA doses, as indicated, and with LPS, serving as positive control, relative to control (*n *= 4). Data are expressed as mean ± SEM. Results were analyzed by Kruskal–Wallis test followed by Dunn’s post hoc test. *P < 0.05; **P < 0.01 vs. control; ^#^*P* < 0.01 vs. *Tlr5*^−*/*−^. **f**, **g** Purified neurons from C57BL/6 mice were incubated with various FLA doses, as indicated, 1 μg/ml LPS, or 1 mM loxoribine (LOX) for 72 h (**f**), or were incubated with 100 ng/ml FLA for indicated durations (**g**, *n* = 3). Subsequently, cells were stained with NeuN antibody and DAPI. NeuN-positive cells were quantified, and results were expressed as relative neuronal viability by setting the viability of control cells to 100%. Data are expressed as mean ± SEM. Results were analyzed by Kruskal–Wallis test followed by Dunn’s post hoc test. *P < 0.05 vs. control; n.s., not significant

Microglia can trigger neurodegeneration through TLRs including TLR2, TLR3, TLR4, TLR7, and TLR9 [6, 30, 36, 38, 40]. To test whether activation of TLR5 in microglia affects neuronal survival, co-cultures of neurons and microglia derived from WT or *Tlr5*^−*/*−^ mice were exposed to flagellin. Assessment of the relative neuronal viability revealed that flagellin induced neuronal loss (Fig. 5a) in a dose (Fig. 5b)- and time (Fig. 5c)-dependent manner. Under control conditions, microglia co-cultured with neurons did not significantly affect neuronal survival, and 1 ng/ml flagellin also had no significant toxic effect within 72 h (loss of NeuN-positive cells by 4.1% (± 1.8, *P* = 0.1443)). In contrast, 10 ng/ml and 100 ng/ml flagellin induced neuronal loss by 19.2% (± 3.4, *P* = 0.017) and 30.9% (± 4.1, *P* = 0.0042), respectively, compared to control condition after 72 h (Fig. 5b). While exposure to 100 ng/ml flagellin did not significantly reduce neuronal viability within 12 h (15.2% ± 2.3, *P* = 0.2177; data not shown), neuronal loss was observed after 1 d, 3 d, and 5 d of flagellin treatment by 26.8% (± 12.0, *P* = 0.0363), 30.9% (± 4.1, *P* = 0.0153), and 32.0% (± 7.9, *P* = 0.0211), compared to control, respectively (Fig. 5c). In accordance with this, flagellin treatment caused an increase in TUNEL-positive cells in neuronal cultures in the presence of WT microglia (Fig. 5d, e). Flagellin-induced neurotoxicity required microglial TLR5, as neurons co-cultured with *Tlr5*^−*/*−^ microglia were protected from flagellin-induced neurotoxicity (Fig. 5a–c). To test whether this neurotoxicity requires microglia, we incubated purified cortical neurons with flagellin, LPS, which induces neuronal injury through microglial TLR4 in co-cultures [40], or loxoribine, an established TLR7 ligand known to induce cell-autonomous neuronal apoptosis [36]. Assessment of the relative neuronal viability revealed that cortical neurons were not affected by flagellin in the absence of microglia during the whole observation period (Fig. 5f, g). Likewise, in contrast to loxoribine, LPS did not affect neuronal survival in cultures of purified neurons, as expected (Fig. 5f).

In summary, these findings demonstrate that activation of TLR5 in microglia results in neuronal apoptosis.

### Intrathecal flagellin induces neuronal injury in the cerebral cortex

**Fig. 6:**
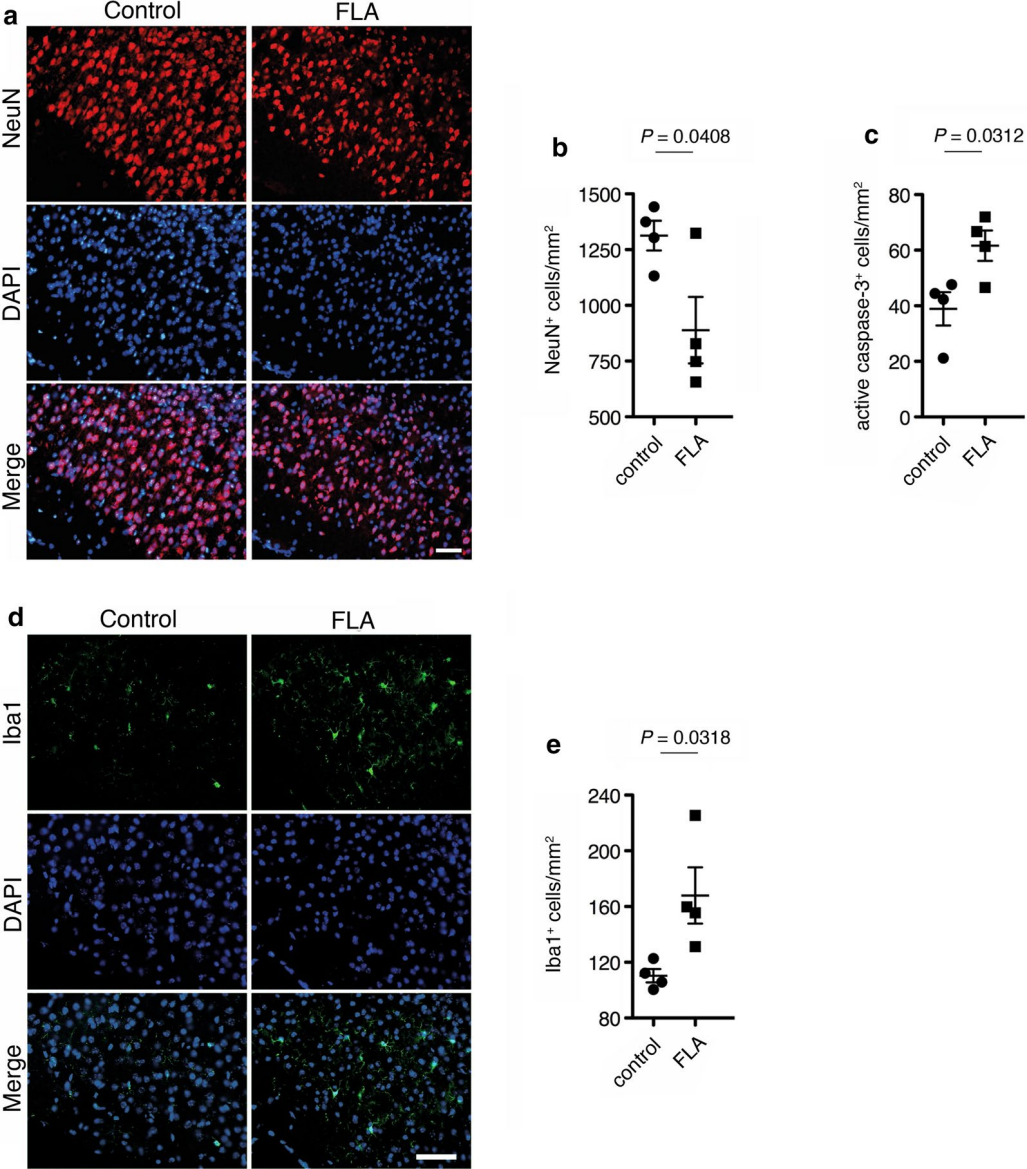
Intrathecal flagellin triggers neuronal injury in the cerebral cortex. 1 μg of flagellin (FLA) was injected intrathecally into C57BL/6 mice (*n* = 4), while PBS served as control (*n* = 4). **a** After 3 d, brain sections were stained with NeuN antibody and DAPI, and representative images are shown (scale bar, 50 μm). **b** The density of NeuN-positive cells in the cerebral cortex was assessed. **c** Sections were stained with active caspase-3 antibody (images are not shown), and caspase-3-positive cells in the cerebral cortex were quantified. **d** Sections were immunostained with Iba1 antibody and DAPI, and **e** Iba1-positive cells in the cerebral cortex were quantified. Results are presented as mean ± SEM. Data were analyzed by Student’s *t*-test and provided indicated *P* values

To evaluate the functional role of flagellin as TLR5 activator in the CNS in vivo, we injected mice intrathecally with flagellin or PBS serving as control. Immunohistochemical analysis of the cerebral cortex 72 h after injection revealed that flagellin induced significant neuronal loss of 32.3% compared to control as revealed by combined NeuN and DAPI labeling (Fig. 6a, b). Induction of apoptosis in the cerebral cortex was confirmed by immunostaining of active caspase 3-positive cells, which were increased by 58.0% in flagellin-injected animals compared to control (Fig. 6c). Furthermore, intrathecal application of flagellin led to increased microglial numbers (52.3%) in the cerebral cortex compared to control condition (Fig. 6d, e).

Taken together, the TLR5 activator flagellin induces neuronal injury in the cerebral cortex in vivo.

## Discussion

Microglia express all TLRs identified so far, and TLR signaling can have a profound impact on microglial function. TLR4 activation by its established ligand LPS, for example, triggers cytokine release from microglia and affects their proliferation [40, 60, 65]. TLR1/2 signaling in microglia promotes a pro-tumorigenic phenotype of these cells [18], whereas TLR2 and TLR7 modulate microglial chemotaxis and cytokine release [29]. Moreover, activation of microglial TLR2, TLR4, and TLR7 contribute to neuronal injury [40, 41]. Although TLR5 expression in human and mouse microglia was previously described [4, 52], and a few studies recently reported on a functional relevance for this receptor in the setting of various CNS disorders including neuropathic pain, stroke, and Alzheimer’s disease (AD) [7, 24, 33, 64], its mode of action and functional consequences of this receptor activation in the brain has not been explored. While for most of the TLRs several agonists derived from pathogens and host-derived tissue were identified [61], the bacterial protein flagellin is the only established natural ligand for TLR5. Yet conversely, flagellin seems to activate further receptor systems, as flagellin from *Legionella pneumophila* triggers the release of proinflammatory molecules such as IL-1β from microglia, through the inflammasome Naip5-NLRC4 complex [32]. However, flagellin from *Salmonella* typhimurium as used in our current study did not result in IL-1β secretion from microglia, suggesting a pathogen-specific activation of TLR5 and subsequent phenotype induction in these cells.

TLR signaling plays a major role in initiating host defense responses in CNS microbial infection. While several TLRs including TLR4, which recognizes Gram-negative bacteria, TLR2, which detects lipoproteins from Gram-positive bacteria, and TLR9 as a sensor for bacterial and viral DNA, were extensively studied in CNS infection [20], data on TLR5 function in this context are rare. Among other TLRs, TLR5 in primate microglia, and also astrocytes, triggers the production of proinflammatory molecules in response to *Borrelia burgdorferi*, thereby possibly contributing to Lyme neuroborreliosis [3]. Furthermore, TLR5 senses flagellin from *Listeria monocytogenes* [22], which represents one of the major pathogens causing bacterial meningitis in humans. In our study, we demonstrate that exposure to flagellin modulates diverse functions of mouse microglia as the brain’s primary immune cells. First, it triggers the release of specific inflammatory molecules, second, it modulates chemotaxis, third, it increases phagocytosis, and finally, it triggers neuronal apoptosis through microglial activation. All these effects require functional TLR5 signaling, as demonstrated in experiments using *Tlr5*^−*/*−^ microglia and/or TLR5-neutralizing antibody. Moreover, our study provides evidence that activation of TLR5 in microglia triggers the PI3K/Akt/mTORC1 pathway, which is required for the different microglial functions described above. Our findings in microglia are in line with studies on peripheral macrophages, in which flagellin triggers the release of TNF-α through PI3K/Akt/mTORC1 signaling [2]. However, the downstream signaling cascade induced by activation of distinct TLRs in microglia seems to involve different molecular components, as PI3K and Rac inhibition impairs both TLR2- and TLR7-mediated microglial migration, whereas Akt phosphorylation is required only for TLR2-, but not for TLR7-induced migration [29]. In peripheral immune cells, the intracellular TLR adapter MyD88 represents a common link for TLR2, TLR5, and TLR7 signaling [35]. Although not investigated in our study, this adapter is likely required in microglial TLR5 signaling as well, as it is crucial for microglial TLR2 and TLR7 signaling [36, 41]. Interestingly, the release of MIP-2 from microglia exposed to LPS was significantly enhanced in the absence of TLR5, while IL-6 and IL-10 responses were reduced compared to wild-type cells. One may speculate that TLR5 cooperates with TLR4 [28], which is the LPS-recognizing receptor, in an unknown manner, resulting in enhancing (IL-6, IL-10) or inhibiting (MIP-2) effects on cytokine production. In addition, as TLR5 can interact with TIR-domain-containing adapter-inducing interferon-β (TRIF), LPS stimulation might modulate the cytokine response through the associated pathway [8]. Furthermore, while microglial production of the particular cytokines/chemokines in response to flagellin was clearly mediated by TLR5 in our study, other receptors and downstream signaling pathways activated by flagellin, which trigger different cytokine responses and subsequently induce a specific microglial phenotype may exist. The observed reduction in the microglial release of cytokines at higher flagellin doses may be explained by the fact that the interaction between TLR5 and flagellin is based on protein structure-mediated receptor-ligand binding at the ratio of 1:1. In addition, flagellin is capable of interacting with itself through the backbone protein [66]. Thus, at 100 ng/ml flagellin saturation of the receptor system in microglia might be achieved, and higher flagellin doses (1000 ng/ml) may form flagellin aggregates, which inhibit further linear activation. Finally, TLR5-mediated signaling in microglia may be negatively regulated at specific flagellin concentrations. In general, protein interactions within the TLR signaling pathway are targeted by one specific or several inhibitors [16], and fine-tuning of the TLR5 signaling pathway might be achieved not only through a cascade of regulators, but also by specific ligand concentrations.

Microglia are activated in essentially all CNS disorders, and the subsequent neuroinflammation, in its characteristic form common to various neurodegenerative diseases, is shaped by their migratory, phagocytic, and antigen-presenting properties. As discussed above, TLRs detect pathogen-associated components, but can also be activated by host-derived factors. For instance, the extracellular matrix component versican activates TLR2, tenascin C and heat-shock protein 60 signal through TLR4, and sequence-specific microRNAs activate TLR7 [5, 17, 27, 36, 41]. Recognition of such endogenous molecules, potentially derived from injured host tissue, by TLRs and the subsequent inflammatory response may have important pathological implications, as both processes contribute to various CNS injury models, including mouse models for various neurodegenerative diseases [41, 53]. Notably, TLR5 specifically recognizes flagellin from bacteria, and bacterial infection has been associated with the increased occurrence of neurodegenerative diseases, such as AD [26, 44, 49]. While in the healthy state, TLR5 expression in mouse and human brain is only weak [21, 42], expression of TLR5 mRNA in the AD cortex is increased and correlates with the expression of the constitutive microglial cell marker Iba1 [7, 24]. Matching our findings in mouse microglia, human THP-1 monocytes exposed to flagellin release cytokines including TNF-α, and this release is prevented by TLR5 inhibition [24]. Consistent reports of increased TLR5 expression exist in the context of further neurodegenerative disorders, such as Parkinson’s disease and dementia with Lewy bodies [14, 43]. We report herein that TLR5 is abundantly expressed in adult microglia whose activation represents a major hallmark in neurodegenerative diseases, and that signaling through this receptor contributes to neuronal apoptosis. Taking into account that TLR5 activation seems to be involved in the different pathological states outlined above, it is conceivable that endogenous, yet unidentified ligands for TLR5 exist. In accordance with this, contribution of TLR5 to further CNS pathologies was recently reported. In neuropathic pain, TLR5 function is involved in mechanical allodynia [64], while in stroked mice, TLR5 activation induces NF-κB via Akt phosphorylation [33], matching our data on flagellin-increased Akt phosphorylation in microglia. Besides injecting mice intrathecally with flagellin as a proof-of-concept demonstration for a role of activated TLR5 in neuronal injury, we herein tested the impact of TLR5 signaling in the setting of glioblastoma, the most aggressive brain tumor in adults. The experiments using the GL261 glioma mouse model were prompted by our previous results on the promotion of tumor growth involving TLR2, TLR4, and TLR7 signaling [5, 12, 27], and were driven by the assumption that TLR5 might detect host-derived molecules. In line with this, high mobility group box 1 was recently suggested to bind to TLR5, thereby activating proinflammatory signaling with the functional consequence of pain [10]. In our experimental glioma set-up, however, neither TLR5 stimulation by flagellin nor deletion of TLR5 signaling as assessed by analyzing *Tlr5*^−*/*−^ mice, had an impact on glioma growth. Therefore, TLRs do not seem to be uniformly involved in the regulation of glioma growth.

## Conclusions

Our study establishes TLR5 and its ligand flagellin as modulators of microglial function and neuronal apoptosis. Further research will be required to investigate the probably more complex role of TLR5 in the CNS and to establish clinical consequences of CNS injury triggered by this receptor in distinct CNS disorders, such as bacterial infection and neurodegenerative diseases. Finally, TLR5 signaling and subsequent regulation of microglial function may be important not only for pathological but also for physiological processes in the CNS.

## Supplementary information

**Additional file 1**: Primary cultures of microglia, astrocytes, and neurons. Microglia, astrocytes, and neurons were isolated from C57BL/6 mice as described in the *Methods* section. Phase contrast images display the respective cell type, as indicated, after 3 d in vitro. Scale bar, 10 μm.

**Additional file 2**: Protein concentrations of cytokines/chemokines released from wild-type and *Tlr5*^−*/*−^ microglia. Multiplex immunoassay was used to detect cytokines/chemokines, as indicated, in supernatants of cultured neonatal microglia from C57BL/6 (wild-type, WT) and *Tlr5*^−*/*−^ mice in response to 100 ng/ml flagellin (FLA) after 24 h. Unstimulated cells served as negative control, while LPS (100 ng/ml) was used as positive control (*n* = 3). Data are expressed in pg/ml ± SD. n.d., not detectable.

**Additional file 3**: Flagellin does not induce microglial injury or cell loss. C57BL/6 microglia were incubated with different flagellin (FLA) doses, as indicated, for 72 h. Unstimulated cells served as negative control. Subsequently, cells were stained with IB4 to mark microglia, TUNEL assay to mark apoptotic cells, and DAPI. (**a**) Representative image of microglia exposed to 5 μg/ml FLA. Scale bar, 50 μm. (**b**) IB4- and (**c**) TUNEL-positive cells were quantified. Results were expressed as absolute number of IB4-positive or TUNEL-positive cells per field (**c**). Data are expressed as mean ± SEM. Results were analyzed by Kruskal–Wallis test followed by Dunn’s post hoc test. n.s., not significant.

**Additional file 4**: Neither TLR5 deficiency nor exposure to flagellin does affect glioma growth ex vivo. (**a**) mCherry GL261 glioma cells were inoculated into organotypic brain slices (OBS) derived from P14-P16 C57BL/6 (*n* = 2; number of inoculated tumors: 27) and P14-P16 *Tlr5*^−/−^ mice (*n* = 3; number of inoculated tumors: 21) and cultured for 4 d. Subsequently, OBS were fixed, stained with Hoechst, and scanned by confocal microscopy, followed by 3D surface reconstruction of gliomas and measurement of tumor volumes. (**b**) OBS derived from P14-P16 C57BL/6 mice were inoculated with mCherry glioma GL261 cells and stimulated with FLA (100 ng/ml, *n* = 3; number of analyzed tumors: 12). Tumor volume was compared to unstimulated control (*n* = 2; number of analyzed tumors: 16). Results are represented as mean ± SEM. Data were analyzed by Student’s *t*-test. n.s., not significant.

## Data Availability

The datasets used and/or analyzed during the current study are available from the corresponding authors on reasonable request.

## Abbreviations

Aβ: beta-amyloid
AD: Alzheimer’s disease
Akt: serine/threonine protein kinase B
CNS: central nervous system
DMSO: dimethyl sulfoxide
FLA: flagellin
IL: interleukin
LPS: lipopolysaccharide
mTORC1: mammalian target of rapamycin complex 1
NeuN: neuronal nuclei
NF-κB: nuclear factor ‘kappa-light-chain-enhancer’ of activated B-cells
NO: nitric oxide
PDK1: 3-phosphoinositide-dependent kinase-1
PI3K: phosphoinositide 3-kinase
Rac: rho family of GTPases
TLR: Toll-like receptor
TUNEL: TdT-mediated dUTP-biotin nick end labeling
