# Supplementary material for: Activation of Toll-like receptor 5 in microglia modulates their function and triggers neuronal injury
Source: Acta Neuropathol Commun. 2020 Sep 10;8:159. doi: 10.1186/s40478-020-01031-3 (PMC7488138; doi:10.1186/s40478-020-01031-3)
Supplement: Supplementary file 1 — Additional file 1: Primary cultures of microglia, astrocytes, and neurons. Microglia, astrocytes, and neurons were isolated from C57BL/6 mice as described in the Methods section. Phase contrast images display the respective cell type, as indicated, after 3 d in vitro. Scale bar, 10 μm. [file 40478_2020_1031_MOESM1_ESM.pdf]

Additional files

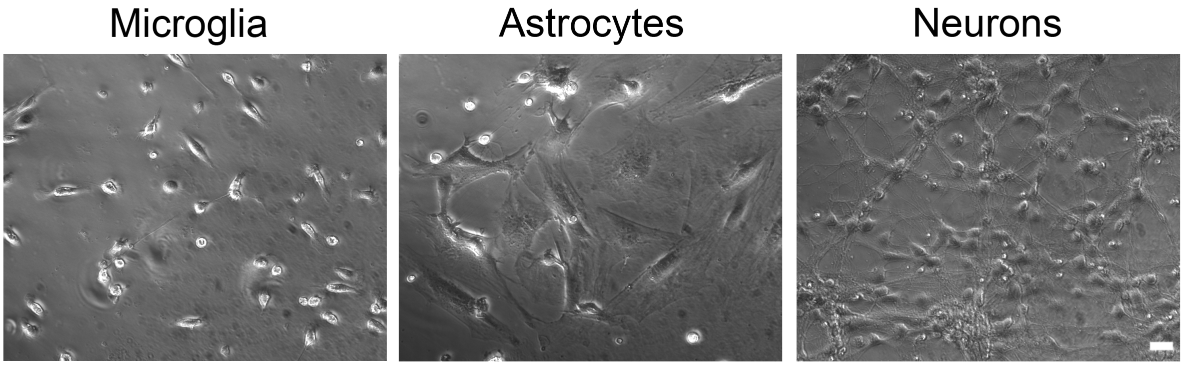

Additional file 1 Primary cultures of microglia, astrocytes, and neurons. Microglia, astrocytes, and neurons were isolated from C57BL/6 mice as described in the *Methods* section. Phase contrast images display the respective cell type, as indicated, after 3 d *in vitro*. Scale bar, 10 μm.
