# Supplementary material for: Activation of Toll-like receptor 5 in microglia modulates their function and triggers neuronal injury
Source: Acta Neuropathol Commun. 2020 Sep 10;8:159. doi: 10.1186/s40478-020-01031-3 (PMC7488138; doi:10.1186/s40478-020-01031-3)
Supplement: Supplementary file 2 — Additional file 2: Protein concentrations of cytokines/chemokines released from wild-type and Tlr5−/− microglia. Multiplex immunoassay was used to detect cytokines/chemokines, as indicated, in supernatants of cultured neonatal microglia from C57BL/6 (wild-type, WT) and Tlr5−/− mice in response to 100 ng/ml flagellin (FLA) after 24 h. Unstimulated cells served as negative control, while LPS (100 ng/ml) was used as positive control (n = 3). Data are expressed in pg/ml ± SD. n.d., not detectable. [file 40478_2020_1031_MOESM2_ESM.pdf]

**WT**	GRO-α	IL-1β	IL-10	IL-6	MIP-2	RANTES	TNF-α
control	20.84 (±21.67)	0.25 (±0.23)	n.d.	n.d.	80.8 (±54.24)	11.63 (±6.14)	22.76 (±16.98)
FLA (100 ng/ml)	177.61 (±102.12)	0.27 (±0.17)	10.25 (±7.07)	13.84 (±9.37)	1,363.56 (±719.79)	379.51 (210.61)	114.54 (±61.69)
LPS (100 ng/ml)	1,231.81 (±418.09)	316.68 (±246.01)	167.47 (±59.67)	17,190.24 (±5,350.14)	824.18 (±122.42)	1,000 (±0)	1,959.46 (±166.81)

Tlr5$^{-/-}$	GRO-α	IL-1β	IL-10	IL-6	MIP-2	RANTES	TNF-α
control	1.00 (±1.74)	n.d.	n.d.	1.13 (±0.23)	7.52 (±4.76)	1.76 (±3.04)	10.35 (±2.14)
FLA (100 ng/ml)	1.02 (±1.76)	n.d.	n.d.	0.84 (±1.07)	9.57 (±6.49)	5.09 (±8.82)	11.47 (±2.40)
LPS (100 ng/ml)	110.10 (±51.40)	n.d.	1.98 (±1.33)	79.54 (±36.88)	1,633.43 (±414.59)	774.15 (±238.01)	636.58 (±142.2)

Additional file 2 Protein concentrations of cytokines/chemokines released from wild-type and *Tlr5*$^{-/-}$ microglia. Multiplex immunoassay was used to detect cytokines/chemokines, as indicated, in supernatant of cultured neonatal microglia from C57BL/6 (wild-type, WT) and *Tlr5*$^{-/-}$ mice in response to 100 ng/ml flagellin (FLA) after 24 h. Unstimulated cells served as negative control, while LPS (100 ng/ml) was used as positive control (*n* = 3). Data are expressed in pg/ml ±SD. n.d., not detectable.
