# Supplementary material for: Activation of Toll-like receptor 5 in microglia modulates their function and triggers neuronal injury
Source: Acta Neuropathol Commun. 2020 Sep 10;8:159. doi: 10.1186/s40478-020-01031-3 (PMC7488138; doi:10.1186/s40478-020-01031-3)
Supplement: Supplementary file 3 — Additional file 3: Flagellin does not induce microglial injury or cell loss. C57BL/6 microglia were incubated with different flagellin (FLA) doses, as indicated, for 72 h. Unstimulated cells served as negative control. Subsequently, cells were stained with IB4 to mark microglia, TUNEL assay to mark apoptotic cells, and DAPI. (a) Representative image of microglia exposed to 5 μg/ml FLA. Scale bar, 50 μm. (b) IB4- and (c) TUNEL-positive cells were quantified. Results were expressed as absolute number of IB4-positive or TUNEL-positive cells per field (c). Data are expressed as mean ± SEM. Results were analyzed by Kruskal–Wallis test followed by Dunn’s post hoc test. n.s., not significant. [file 40478_2020_1031_MOESM3_ESM.pdf]

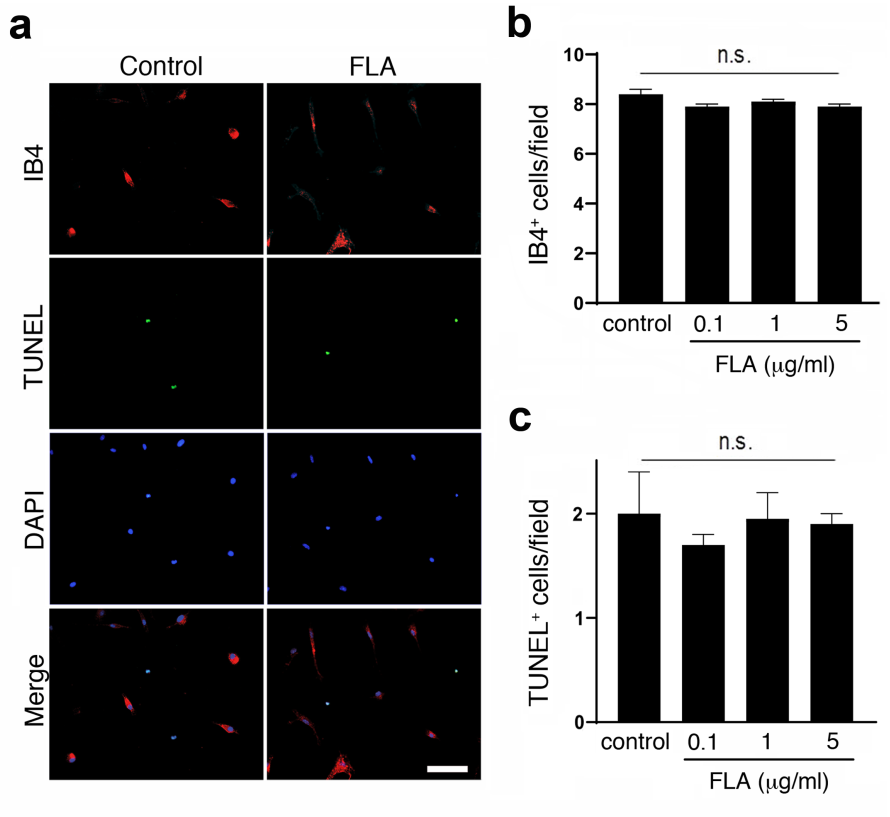

Additional file 3 Flagellin does not induce microglial injury or cell loss. C57BL/6 microglia were incubated with different flagellin (FLA) doses, as indicated, for 72 h. Unstimulated cells served as negative control. Subsequently, cells were stained with IB4 to mark microglia, TUNEL assay to mark apoptotic cells, and DAPI. (**a**) Representative image of microglia exposed to 5 μg/ml FLA. Scale bar, 50 μm. (**b**) IB4- and (**c**) TUNEL-positive cells were quantified, and results were expressed as absolute number of IB4- or TUNEL-positive cells per field. Data are expressed as mean ± SEM. Results were analyzed by Kruskal-Wallis test followed by Dunn's post hoc test. n.s., not significant.
