# Supplementary material for: Activation of Toll-like receptor 5 in microglia modulates their function and triggers neuronal injury
Source: Acta Neuropathol Commun. 2020 Sep 10;8:159. doi: 10.1186/s40478-020-01031-3 (PMC7488138; doi:10.1186/s40478-020-01031-3)
Supplement: Supplementary file 4 — Additional file 4: Neither TLR5 deficiency nor exposure to flagellin does affect glioma growth ex vivo. (a) mCherry GL261 glioma cells were inoculated into organotypic brain slices (OBS) derived from P14-P16 C57BL/6 (n = 2; number of inoculated tumors: 27) and P14-P16 Tlr5−/− mice (n = 3; number of inoculated tumors: 21) and cultured for 4 d. Subsequently, OBS were fixed, stained with Hoechst, and scanned by confocal microscopy, followed by 3D surface reconstruction of gliomas and measurement of tumor volumes. (b) OBS derived from P14-P16 C57BL/6 mice were inoculated with mCherry glioma GL261 cells and stimulated with FLA (100 ng/ml, n = 3; number of analyzed tumors: 12). Tumor volume was compared to unstimulated control (n = 2; number of analyzed tumors: 16). Results are represented as mean ± SEM. Data were analyzed by Student’s t-test. n.s., not significant. [file 40478_2020_1031_MOESM4_ESM.pdf]

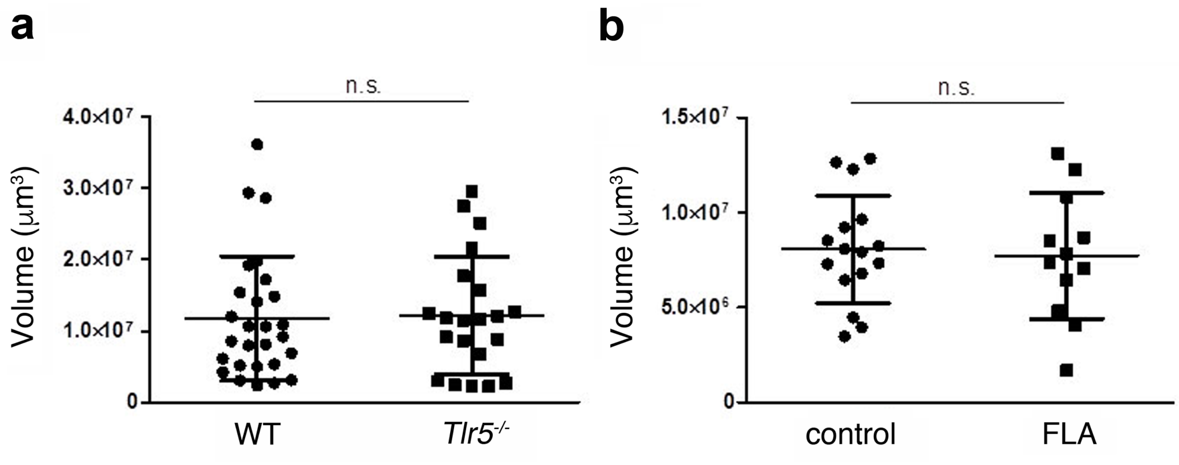

Additional file 4 Neither TLR5 deficiency nor exposure to flagellin does affect glioma growth *ex vivo*. (**a**) mCherry GL261 glioma cells were inoculated into organotypic brain slices (OBS) derived from P14-P16 C57BL/6 (n = 2; number of inoculated tumors: 27) and P14-P16 *Tlr5*$^{-/-}$ mice (n = 3; number of inoculated tumors: 21) and cultured for 4 d. Subsequently, OBS were fixed, stained with Hoechst, and scanned by confocal microscopy, followed by 3D surface reconstruction of gliomas and measurement of tumor volumes. (**b**) OBS derived from P14-P16 C57BL/6 mice were inoculated with mCherry glioma GL261 cells and stimulated with FLA (100 ng/ml, n = 3; number of analyzed tumors: 12). Tumor volume was compared to unstimulated control (n = 2; number of analyzed tumors: 16). Results are represented as mean ± SEM. Data were analyzed by Student's *t*-test. n.s., not significant.
